# Editorial: Advances in understanding and managing systemic sclerosis-associated interstitial lung disease: bridging prognostic biomarkers to therapeutic innovations

**DOI:** 10.3389/fmed.2025.1683554

**Published:** 2025-09-16

**Authors:** Gonçalo Boleto, Tânia Santiago, Cristiana Sieiro Santos

**Affiliations:** ^1^Rheumatology Department, Hospital de Santa Maria, U.L.S. Santa Maria, Lisbon, Portugal; ^2^Faculdade de Medicina, Universidade de Lisboa, Centro Académico de Medicina de Lisboa, Lisbon, Portugal; ^3^Rheumatology Department, U.L.S. de Coimbra, Coimbra, Portugal; ^4^Medicine Faculty, University of Coimbra, Coimbra, Portugal; ^5^Centre for Musculoskeletal Research, School of Biological Sciences, Faculty of Biology, Medicine and Health, University of Manchester, Manchester, United Kingdom

**Keywords:** scleroderma, lung disease, biomarkers, prognosis, interstitial lung disease

Systemic sclerosis (SSc) is a rare and complex autoimmune connective tissue disease characterized by immune dysfunction, excessive fibrotic tissue deposition, and progressive microvascular injury. Interstitial lung disease (ILD), affecting approximately 40% of SSc patients, is a leading cause of morbidity and accounts for 10%−20% of SSc-related deaths (1).

In the largest study using data from the European Scleroderma Trials and Research Group (EUSTAR) database to analyze progressive ILD (2), 27% of patients with SSc-associated ILD (SSc-ILD) met the criteria for progression, defined as a decline in FVC ≥10% or a 5%−10% decline in FVC with a ≥15% decrease in DLCO. Notably, patterns of progression were inconsistent across annual intervals: many patients with overall FVC decline experienced periods of stability or improvement, while others with overall improvement had intermittent declines. These findings underscore the urgent need to identify patients at high risk of irreversible pulmonary damage early in the disease course (3). Yet, reliable prognostic tools for stratifying risk and guiding personalized therapy remain limited.

Established clinical risk factors for severe progressive SSc-ILD include diffuse cutaneous involvement, anti-topoisomerase I antibodies (anti-Scl70), elevated acute phase reactants, and early declines in FVC and/or DLCO (4). However, these features alone are insufficient to accurately predict disease trajectories at an individual level. As a result, there is increasing interest in refining clinical phenotyping through the incorporation of novel circulating biomarkers and advanced computational tools. This has spurred a growing effort to identify serum biomarkers that can aid in risk stratification, anticipate disease progression, and monitor treatment response in connective tissue disease-associated ILD. In parallel, computational approaches such as machine learning are being explored to enhance individualized risk prediction and integrate biomarker data with clinical and imaging parameters.

In this Research Topic, we aimed to highlight emerging contributions that advance our understanding of SSc-ILD pathogenesis, prognosis, and potential biomarkers of disease progression.

One of the studies explored the role of extracellular vesicles (EVs), membrane-bound particles derived from endothelial cells, platelets, and leukocytes, which have been implicated in vasculopathy, inflammation, coagulation, and potentially fibrosis in SSc (5). Colic et al. analyzed 32 patients with SSc-ILD and found significantly higher circulating EV levels—measured by flow cytometry—compared to SSc patients without ILD and to controls. Higher EV levels correlated with lower FVC and DLCO and with the presence of respiratory symptoms. EVs were also independent predictors of ILD progression, defined by a decline in FVC ≥10%. Although further validation in larger cohorts is needed, EVs may represent a promising biomarker for early identification of patients at risk of progression and likely to benefit from timely immunomodulatory therapy.

In parallel, the intersection between metabolic dysfunction and pulmonary decline has garnered attention. Yang et al. investigated this association using the atherogenic index of plasma (AIP)—the ratio of triglycerides to HDL cholesterol—which reflects both atherogenic propensity and systemic inflammation. Analyzing data from 4,565 participants in the National Health and Nutrition Examination Survey (NHANES), the authors found a significant inverse correlation between AIP and lung function parameters (FVC, FEV1, FEF25–75%), especially in men and individuals under 40. In a machine learning regression model, HDL-C was the strongest contributor, followed by triglycerides. Although not specific to SSc, these findings support a possible indirect role for AIP and lipid metabolism in ILD evaluation. Previous studies further reinforce this link, showing ILD as a risk factor for ischemic heart disease (6) and a high prevalence of peripheral and carotid artery disease in ILD patients (7). Given the elevated cardiovascular risk in SSc, exploring AIP in SSc-ILD may bring important insights.

Another study by Cano-García et al. assessed the utility of C-reactive protein (CRP) as a biomarker of SSc-ILD and lung damage in comparison to standard blood count based inflammatory indices. CRP and other acute phase reactants (e.g., neutrophil-to-lymphocyte ratio) are inexpensive and widely available biomarkers. In a cohort of 83 SSc patients, including 28 with SSc-ILD, a CRP-based inflammatory cluster was associated with ILD and showed an inverse correlation with FVC. This cluster outperformed hematological indices in predicting lung involvement. The association was stronger in patients positive for anti-topoisomerase I antibodies and those on immunosuppressants. Although CRP levels are not typically elevated in SSc, previous studies and EUSTAR data have linked persistent CRP elevation to ILD and greater disease severity (8). These findings highlight CRP as a potentially valuable marker for early identification and risk stratification in SSc-ILD.

From a diagnostic and phenotypic standpoint, SSc and mixed connective tissue disease (MCTD) share overlapping features in cardiopulmonary involvement, posing challenges for differential diagnosis and tailored management. Pulmonary function tests (PFTs) remain essential in ILD evaluation in both conditions (9, 10). Zhou et al. compared cardiopulmonary features in 138 SSc and 56 MCTD patients. The prevalence of ILD was non-significantly different (17.4% in SSc vs. 21.4% in MCTD), but the authors observed key differences in PFTs: while FVC levels were comparable, SSc patients had lower FEF50 values, suggesting small airway dysfunction, whereas MCTD patients had lower total lung capacity (TLC) and residual volumes (RV). These results reinforce the value of PFTs not only for disease monitoring but also for distinguishing phenotypic patterns relevant for personalized management.

In conclusion, early recognition of preclinical and progressive SSc-ILD remains a pressing unmet need, particularly as therapeutic options with potential to alter disease trajectories become widely available. Accurate risk stratification remains a central priority in SSc research. The studies included in this Topic provide promising insights into novel biomarkers—ranging from EVs to CRP and lipid metabolism—that may improve early detection, guide treatment decisions, and ultimately reduce SSc-related morbidity and mortality. Future directions should prioritize external validation in multicenter cohorts, integration of biomarker panels into routine clinical algorithms, and incorporation into prospective clinical trials aimed at improving long-term outcomes in SSc-ILD.
